# Effects of reminiscence therapy on psychological outcome among older adults without obvious cognitive impairment: A systematic review and meta-analysis

**DOI:** 10.3389/fpsyt.2023.1139700

**Published:** 2023-03-30

**Authors:** Lijun Xu, Shasha Li, Renfu Yan, Yingyuan Ni, Yuecong Wang, Yue Li

**Affiliations:** ^1^Department of Nursing, College of Medical Science, Huzhou University, Huzhou, Zhejiang, China; ^2^Department of Neurosurgery, Huzhou Central Hospital, Huzhou, Zhejiang, China

**Keywords:** reminiscence therapy, older adults, psychosocial outcomes, depression, life satisfaction, meta-analysis

## Abstract

**Introduction:**

Reminiscence therapy has been a high-benefit and low-cost measure of psychosocial intervention for older adults in recent years. It has attracted much attention in the intervention study of older adults without obvious cognitive impairment. This study aimed to evaluate the effects of reminiscence therapy on psychosocial outcomes among older adults without obvious cognitive impairment and analyze the divergences of different intervention programs (form, duration, and setting) on outcomes.

**Methods:**

We searched the commonly used databases and used RevMan 5.4 in the meta-analysis (PROSPERO-ID: CRD42022315237). All eligible trials used the Cochrane Risk of Bias Tool and the Effective Public Health Practice Project quality assessment tool to identify the quality and determine the bias risk grade.

**Results:**

Twenty-seven studies were included, involving 1,755 older adults. Meta-analysis showed that reminiscence therapy has a significant effect on both depression and life satisfaction. Group reminiscence played a significant role in improving life satisfaction. Depression symptoms were not affected by the intervention duration (*P* = 0.06), while life satisfaction was significantly improved after more than 8 weeks of intervention (*P* < 0.00001). Intervention settings drove differences in depressive symptoms (*P* = 0.02), and the effect size of the community was larger.

**Conclusion:**

Reminiscence therapy can significantly reduce depressive symptoms and improve life satisfaction. There are different effects of reminiscence therapy in different intervention schemes on psychological outcomes among older adults. More well-designed trials with large sample sizes and long-term follow-ups are necessary to confirm and expand the present results.

**Systematic review registration:**

https://www.crd.york.ac.uk/prospero/display_record.php?RecordID=315237, identifier: CRD42022315237.

## 1. Introduction

With the decline in the fertility rate, weakened family function, and imperfect social support, the psychosocial health of older adults has become a global public health problem (1, 2). A systematic review showed that the improvement of social and psychological outcomes of older adults with non-obvious cognitive impairment is the decisive factor for active aging (3). Research has confirmed that psychological interventions can improve the social and psychological wellbeing of older adults (4). Therefore, systematic analysis of psychological intervention studies on older adults has attracted the attention of researchers.

Reminiscence therapy has been a high-benefit and low-cost measure of psychosocial intervention for older adults in recent years (5). The concept of reminiscence therapy is that people review and reflect on significant personal past experiences to promote pleasure, better quality of life, and adaptability (6). It mainly includes instrumental reminiscence, spiritual reminiscence, transmissive reminiscence, and integrative reminiscence (7). Existing reminiscence therapy intervention programs are designed in various forms and types of combinations. Shellman et al. (8) adopted individual integrative reminiscence therapy to alleviate depression in older adults by recalling positive events and transferring negative thinking. Meléndez-Moral et al. (9) used group instrumental reminiscence therapy to improve the psychological resilience and adaptability of older adults by reviewing life experiences and analyzing effective strategies. Analyzing literature found that the existing reminiscence therapy has intervened in the psychosocial outcome indicators of older adults through various forms and types of combinations, and has been widely explored in the promotion of the psychological wellbeing of older adults.

As a means of psychological intervention, reminiscence therapy is applied in the form of individual and group therapy (10, 11). Amieva et al. (12) applied reminiscence therapy in group sessions, which did not significantly improve individual cognitive function, depression, or other outcomes, and noted that individualized intervention should be used for older adults. When comparing the effects of individual and group reminiscence therapy on depression in older adults, Bai and Shen (13) found that reminiscence therapy could effectively reduce depression, and individual reminiscence therapy was less effective than group reminiscence therapy on depression remission. Furthermore, Roback (10) evaluated the adverse consequences of group reminiscence therapy in terms of privacy, individualized management, therapist attributes, and risk factors. Although the effects of group and individual reminiscence therapy on different psychological outcomes in older adults have been extensively studied, the choice of form for intervention remains controversial.

Woods et al. (14) have shown that the effects of reminiscence therapy can depend on the differences in the setting and duration of intervention. Chao et al. (15) organized 12 nursing home older adults to participate in reminiscence therapy once a week for an hour, for a total of nine sessions. Results show older adults in reminiscence group did not significantly reduce depression or improve life satisfaction. Choy and Lou (16) invited 46 older adult community residents to carry out reminiscence therapy once a week, one and a half hours each, for a total of six sessions. The results show that the depression level of older adults was significantly reduced, but no improvement in life satisfaction was found in the post-test. Thus, due to the diversity of program designs in reminiscence therapy, there are differences in the improvement of psycho-social outcomes among older adults without significant cognitive impairment.

At present, the design and implementation of reminiscence therapy programs are usually completed based on literature or experience (17–19), but the small sample size limits the promotion and reliability of intervention design (20). Meta-analysis becomes a powerful way to pool data to find the best intervention design. A recent systematic review and meta-analysis evaluated the effects of a reminiscence-based intervention on a wide range of psychosocial outcomes in older adults without cognitive impairment, with subgroup analyses of intervention forms (21). Results demonstrated the effectiveness of the intervention but did not show differences across intervention forms. However, a study of life review intervention for older adults conducted a subgroup analysis of the intervention design and indicated that depression in older adults was markedly reduced only when the duration of the intervention was >8 weeks (22). Therefore, the optimal intervention design for reminiscence therapy and the differences in psychological outcomes between diversity intervention designs are unknown. There is an urgent need to explore specific interventions for reminiscence therapy in older adults through meta-analyses.

Based on the uncertainty of current reminiscence therapy on the psychological outcome indicators among older adults and the important role of intervention implementation (7). The aim of this study was to through systematic evaluation and meta-analysis evaluate the effect of reminiscence therapy on psychosocial outcome indicators among older adults without obvious cognitive impairment and explore the effect of different reminiscence therapy intervention programs (e.g., intervention form, duration, and setting) in older adults without obvious cognitive impairment. The study not only provides theoretical support for reminiscence therapy for old adults but also provides an evidence-based basis for health promotion workers to implement the intervention with reminiscence therapy. It will be conducive to the in-depth implementation of social and psychological health promotion for older adults.

## 2. Method

This review was carried out and reported according to the Preferred Reporting Items for Systematic Reviews and Meta-Analyses (PRISMA) guidelines. This systematic review has been registered in PROSPERO with the number CRD42022315237.

### 2.1. Inclusion and exclusion criteria

Studies were included as follows: (1) Randomized controlled trials (RCT) or quasi-experimental studies; (2) Participants (≥60 years) were cognitively intact or without significant cognitive impairment as well as depression, memory loss, suicidal tendencies or acute mental disorders (e.g., Mini-Mental Status Exam score of 21 or higher); (3) Any form of reminiscence therapy compared with routine nursing or other types of intervention; (4) Studies reporting any of the following psychosocial-related outcomes: depression, loneliness, anxiety, self-esteem, and hopelessness. Studies were excluded if they met the following criteria: (1) Observational studies and other types of studies, such as case reports, commentaries, literature reviews; (2) Older adults with impaired cognition or dementia or depression or other psychiatric disorders; (3) Participants were also receiving other types of interventions (e.g., reminiscence mixed physical exercises or clinical treatments) that could interfere with the outcomes; (4) The whole quality of the study was weak.

### 2.2. Literature search and selection

English databases (such as PubMed, EMBASE, Ovid, CINHAL, MEDLINE) and Chinese databases (CNKI and Wanfang Data) were searched from inception until Feb 1st, 2022 using the following search terms: “aged (Mesh)” OR “old people” OR “older adult^*^” OR “elderly” AND “reminiscence” OR “reminiscence therapy” OR “reminiscence intervention” NOT “dementia” OR “Alzheimer's”. This review focused on psychosocial well-being, but psychological results were not included in the search terms to ensure that as many relevant studies reporting broad psychological results were found as possible. Additional articles were manually identified from the reference lists of the retrieved articles. Two researchers independently screened the literature back-to-back according to the inclusion and exclusion criteria. Divergences were resolved through discussion with a third reviewer.

### 2.3. Quality assessment and data extraction

The quality and the bias risk grade of all eligible trials were identified or determined by the Cochrane Risk of Bias Tool (23). Its evaluation principles include six aspects: selection bias, implementation bias, measurement bias, data bias, publication bias and other bias. The Effective Public Health Practice Project (EPHPP) quality assessment tool was selected to assess the quasi-experimental study, and it includes six aspects and three ratings (24). It was designed for non-randomized intervention studies and has been shown adequate content validity (25). Two reviewers assessed all included studies independently, and disagreements were resolved through discussion with the third reviewer. The final results are shown in the graphs generated by Review Manager 5.4. The following data were extracted from the studies: the first author, year of publication, country, proportion of females and focused on study designs, especially intervention contents, duration, frequency and outcomes.

### 2.4. Statistical analysis

Data analyses were conducted in Review Manager Software. For continuous variables, the mean difference (MD) and standardized mean difference (SMD) were selected as the summary measures for the same outcome measured by the same or different scales, respectively. Heterogeneity was quantified using the χ^2^-test and *I*^2^ statistic. Generally, *p* > 0.1 and *I*^2^ <50% suggested that there was no significant heterogeneity; thus, a fixed-effect model was used. Otherwise, a random-effects model was used. When heterogeneity was too large to combine, we solved it by narrative descriptions. In addition, when more than 10 studies were included in the meta-analysis, funnel charts were used to assess publication bias (23). The data were checked and entered by two researchers, and the level of significance was taken as 5%. In this review, 17 studies provided complete data for analysis. However, due to differences in statistical methods, 10 studies did not present the data we needed (mean, SD). We converted the study data of Cook (26) through the formula recommended by the Cochrane handbook. Predefined subgroup analysis was based on the different characteristics of the intervention program (i.e., forms, durations, settings, and follow-up time), and the primary outcomes were summarized and presented.

## 3. Results

### 3.1. Literature search results

**Figure 1:**
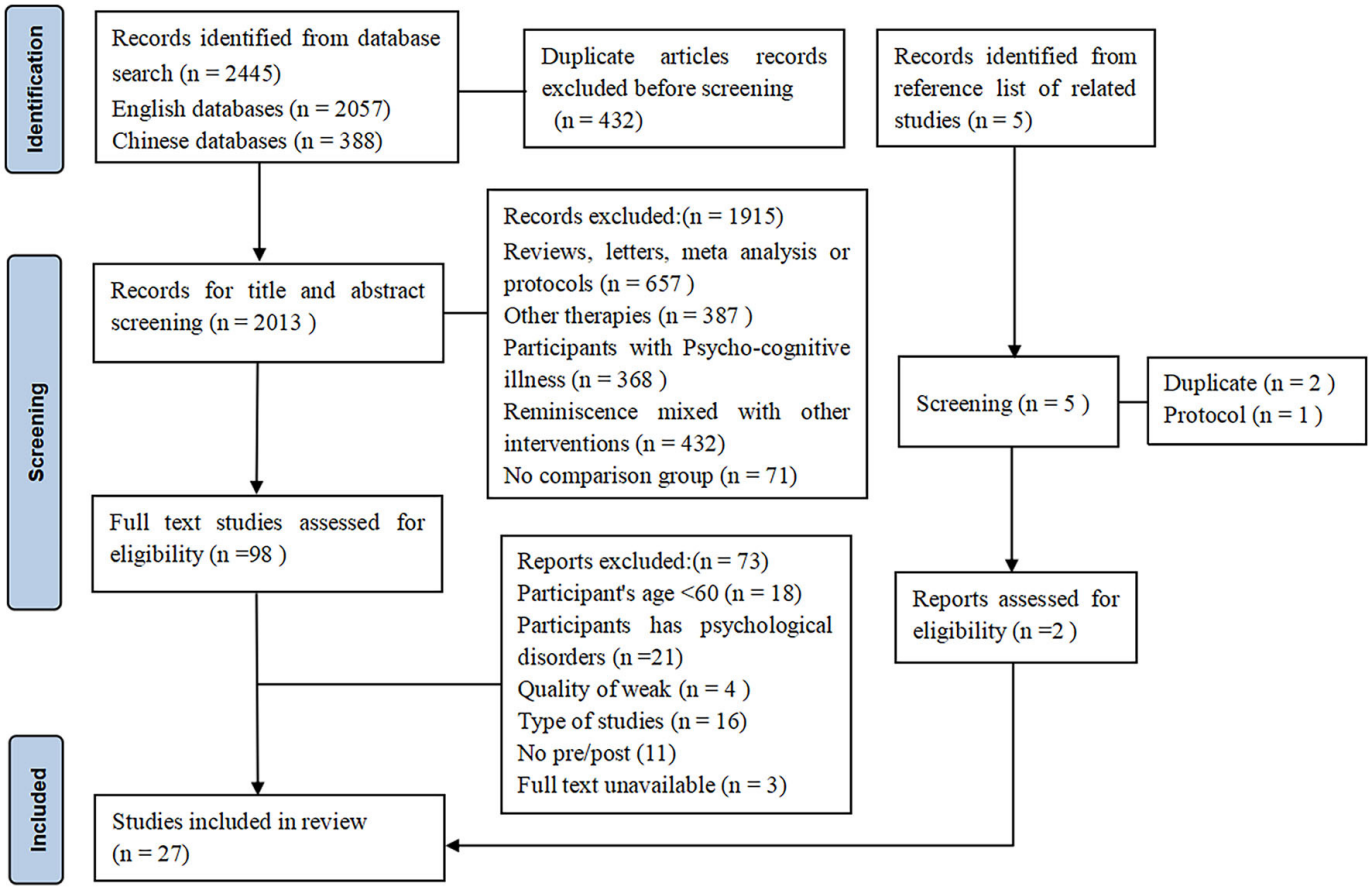
PRISMA diagram summarizing the records retrieval and workflow.

A total of 2,445 records were initially retrieved. A total of 432 duplicate records were excluded. Subsequently, after filtering by title and abstract, 1.915 records were excluded. The remaining 98 studies were fully reviewed, and 73 studies did not meet the inclusion criteria. In addition, 5 studies were obtained from relevant research references. Two studies were included after a thorough review. Finally, 27 studies were included, including 21 randomized controlled trials (RCTs) and 6 quasi-experimental studies. Figure 1 shows a detailed screening process.

### 3.2. Study characteristics

Characteristics of the included studies were shown in Supplementary Table 1. A total of 27 studies were reviewed, including 1,755 older adults. The sample size ranged from 24 to 168. Of these studies, six (8, 17, 26–29) were from the United States, three (18, 19, 30) were from Turkey, three (9, 31, 32) were from the Dominican Republic, three (33–35) were from Iran, two (36, 37) were from Spain, three were from Malaysia (38), Australia (39) and Korea (40), and seven (15, 16, 41–45) were from China. The average age ranged from 65.7 to 84.8. Most studies (*n* = 20) involved mixed gender groups, four studies included only female participants, and three studies recruited only male participants. Eighteen studies provided intervention in the form of groups, while 9 studies provided interventions in the form of individual sessions. The range of intervention sessions ranged from 3 to 16, mostly once a week, 3 twice a week and 1 once a month. The duration of intervention ranged from 2 weeks to 16 weeks, and each duration ranged from 20 min to 4 h. Fourteen studies involved post-intervention follow-up, ranging from 1 day to 6 months. In addition, the instructors varied among the studies, including psychologists, nurses and health specialists, students, trainers and unidentified personnel. The more common indicators are depression, anxiety, life satisfaction, and so on.

### 3.3. Study quality

**Figure 2:**
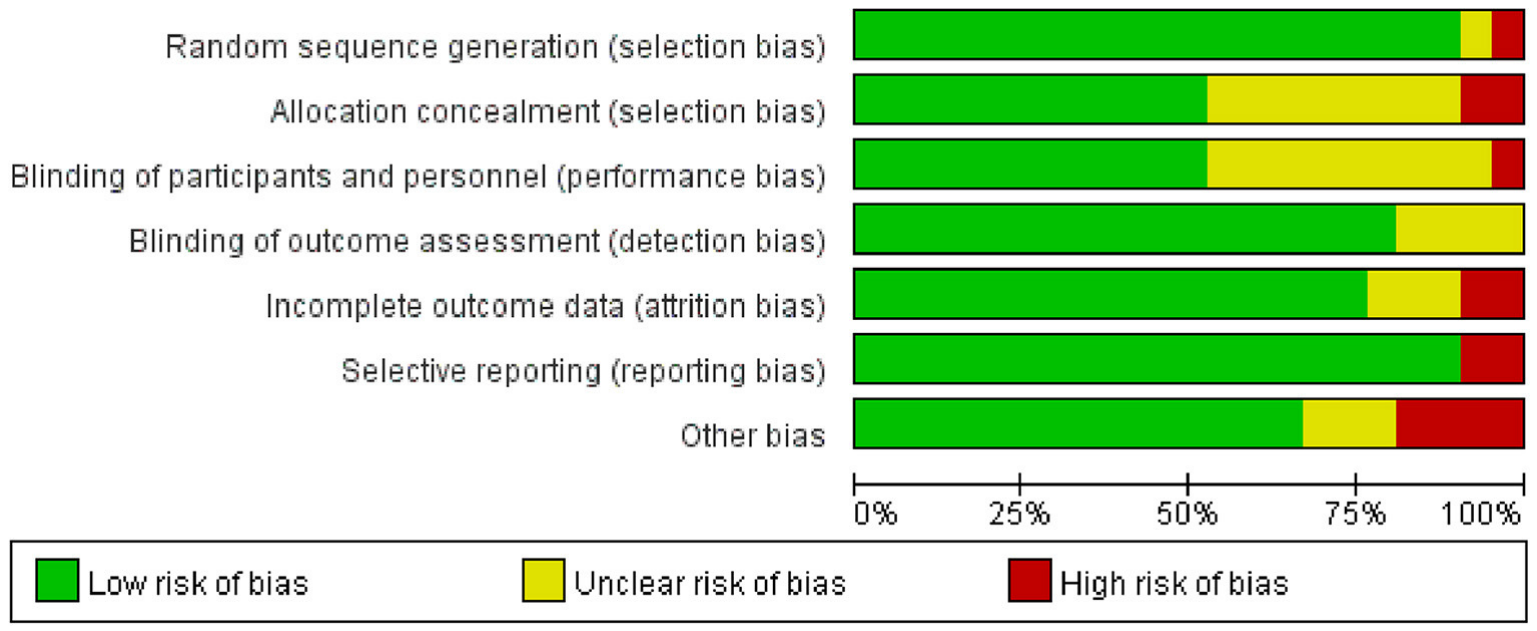
Risk of bias graph of RCTs.

Of the 21 RCTs included, more than 75% presented a low risk of bias in the randomized sequence generation, blinding of outcome assessment, and selective reporting. The outcomes of over 60% of the included studies were not affected by the blinding of participants and personnel, and complete data were reported, without any other significant potential bias. However, the specific process of allocation concealment was not clearly described in 43% of the studies (Figure 2). In addition, six quasi-experimental studies were rated as moderate or strong in quality, and three studies were weak on blinded evaluations (Table 1).

**Table 1:** Quality evaluation of quasi-experimental studies.

**Author/year**	**Selection bias**	**Study design**	**Confounders**	**Blinding**	**Data collection methods**	**Withdrawals and dropouts**	**Global rating**
Chao et al. (15)	M	S	M	W	S	S	M
Ligon (27)	M	M	S	M	S	S	S
Meléndez Moral et al. (36)	M	S	M	W	S	M	M
Sok (40)	S	S	M	W	S	S	M
Saredakis et al. (39)	M	S	M	M	M	M	S
Wu (41)	M	S	M	M	S	S	S

S, strong; M, moderate; W, weak.

### 3.4. Results of psychosocial outcomes

#### 3.4.1. Depression

**Figure 3:**
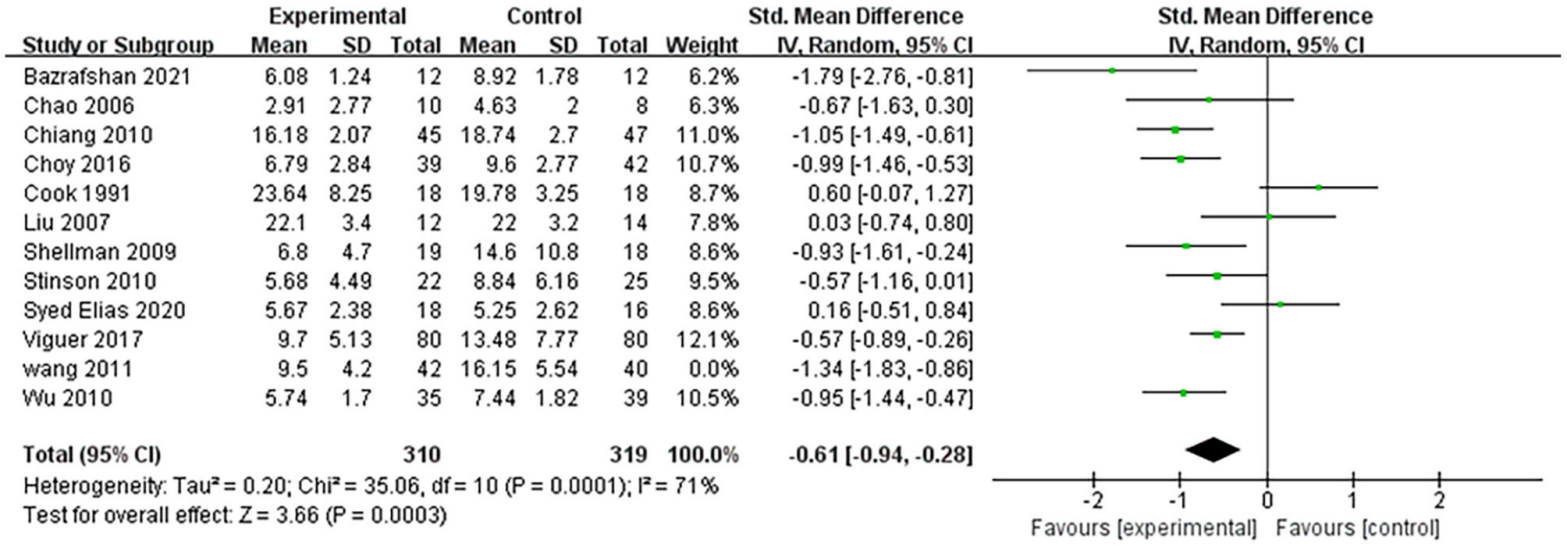
Effect size of the reminiscence therapy group vs. the control group on depression rating scores.

A total of 15 studies reported depression as an outcome, and only 12 of them provided sufficient data for a meta-analysis. The pooled data indicated that the experimental group with reminiscence therapy had a significant effect compared to the control group (SMD: −0.61, 95% CI: −0.94, −0.28). This provides support for reminiscence therapy to effectively relieve the depressive symptoms of older adults (Figure 3).

#### 3.4.2. Life satisfaction

**Figure 4:**
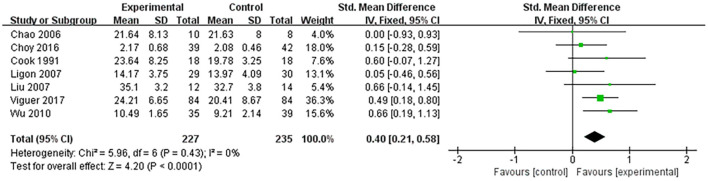
Effect size of the reminiscence therapy group vs. the control group on life satisfaction rating scores.

Nine studies assessed the effect of life satisfaction on older adults and seven studies including 462 participants provided sufficient data. The pooled data showed a significant difference in the improvement of life satisfaction between the reminiscence therapy and control groups (SMD: 0.40, 95% CI: 0.21, 0.58). For the remaining two studies (28, 36) without sufficient data, both reported significant increases in life satisfaction. Therefore, it could be said that reminiscence therapy plays a significant role in the improvement of life satisfaction in older adults (Figure 4).

#### 3.4.3. Self-esteem

Six studies assessed the effect of reminiscence on self-esteem, but one (36) did not provide definitive data. Although the heterogeneity of these five analyzable studies was extremely high (I^2^ = 92%), five studies all reported markedly improvements in self-esteem. One study (26) found that the participants in the reminiscence group scored higher on the mean than the control group (Supplementary Figure 1).

#### 3.4.4. Loneliness

Four studies assessed the effect of reminiscence on loneliness, and all provided sufficient analyzable data. Although the heterogeneity was significantly high (*I*^2^ = 97%), all four studies reported a significant decrease in the mean score of posttest loneliness. In addition, three studies measured loneliness scores after 3 months of follow-up, two studies (42, 43) found a decrease in loneliness, and the study by Syed Elias et al. (38) found no significant difference (Supplementary Figure 1).

#### 3.4.4. Anxiety

Three studies assessed the effect of reminiscence on anxiety. After summarizing the data, it was found that the heterogeneity was noticeably high (*I*^2^ = 95%). Of the three studies, two found (33, 35) a significant decrease in anxiety levels compared to the control group, while one study (38) found a decrease in anxiety scores on the posttest, but the difference was not statistically significant (Supplementary Figure 1).

#### 3.4.5. Happiness

Two studies assessed the effect of reminiscence on happiness. One study (34) explored the influence of narrative reminiscence on the happiness of elderly women, and the results showed no significant difference compared with the control group, while another study (44) found a significant increase in happiness for empty-nest older adults (Supplementary Figure 1).

### 3.5. Results of subgroup and sensitivity analysis

We made a subgroup on depression and life satisfaction, and conducted a sensitivity analysis for depression because it included more than 10 studies. The preset subgroup analysis was based on the different characteristics of the intervention, i.e., forms, durations, settings and follow-up time of the intervention. However, only depression had sufficient post follow-up data, but we abandoned it due to large heterogeneity in both subgroups (*I*^2^ = 85%, *I*^2^ = 93%).

Among the pooled results of depression subgroup comparisons, there were statistically significant differences in the form and setting of the intervention but no significant differences were found in duration (P = 0.06). Subgroups of individual sessions (SMD: −1.21; 95% CI: −1.60, −0.81) and community (SMD: −1.21; 95% CI: −1.42, −0.82) showed larger effects. However, from subgroup comparisons of aggregated life satisfaction results, we found significant differences in the forms, duration and settings of the intervention. Compared with individual sessions, group sessions were statistically significant (SMD: 0.45; 95% CI: 0.25, 0.65). The intervention effect did not appear to be significant when the intervention time was −8 weeks (SMD: 0.11; 95% CI: −0.22, 0.44), whereas reminiscence was more effective when the duration was longer than 8 weeks (SMD: 0.53; 95% CI: 0.30, 0.75). Furthermore, we found significant differences in different environments of intervention. The results showed that reminiscence had no effect on older adults in the community (SMD: 0.11; 95% CI: −0.22, 0.44) but had significance on the residents of care homes (SMD: 0.53; 95% CI: 0.30, 0.75). Main results were summarized in Tables 2, 3.

**Table 2:** Results of subgroup analysis of depression.

**Subgroups**	***I* ^2^**	**Studies (*n*)**	**Participants (*n*)**	**SMD**	**95% CI**	***P* (overall effects)**	***P* (group differences)**
**Form of intervention**							*P* = 0.02
Individual sessions	0%	2	119	−1.21	−1.60, −0.81	*P* < 0.00001	
Group sessions	74%	10	592	−0.58	−0.93, −0.23	*P* = 0.001	
**Duration of intervention**							*P* = 0.06
<8weeks	77%	4	186	−0.75	−1.41, −0.08	*P* = 0.03	
=8weeks	0%	3	211	−1.14	−1.43, −0.84	*P* < 0.00001	
>8weeks	36%	5	314	−0.51	−0.84, −0.18	*P* = 0.003	
**Setting of intervention**							*P* = 0.02
Care home	63%	9	511	−0.60	−0.92, −0.28	*P* = 0.0003	
Community	0%	3	200	−1.12	−1.42, −0.82	*P* < 0.00001	

*I*^2^, heterogeneity; SMD, standardized mean difference.

**Table 3:** Results of subgroup analysis of life satisfaction.

**Subgroups**	***I* ^2^**	**Studies (n)**	**Participants (n)**	**SMD**	**95% CI**	***P* (overall effects)**	***P* (group differences)**
**Form of intervention**							*P* = 0.15
Individual sessions	0%	1	59	0.05	−0.46, 0.56	*P* = 0.85	
Group sessions	0%	6	403	0.45	0.25, 0.65	*P* < 0.00001	
**Duration of intervention**							*P* = 0.04
<8weeks	0%	2	140	0.11	−0.22, 0.44	*P* = 0.51	
>8weeks	0%	5	322	0.53	0.30, 0.75	*P* < 0.00001	
**Setting of intervention**							*P* = 0.04
Care home	0%	5	322	0.53	0.30, 0.75	*P* < 0.00001	
Community	0%	2	140	0.11	−0.22, 0.44	*P* = 0.51	

I^2^, heterogeneity; SMD, standardized mean difference.

In addition, sensitivity analyses for depression showed that excluding any study individually had no substantial effect on SMD on a scale of −0.79 (95% CI: −1.07, −0.52) to −0.61 (95% CI: −0.94, −0.28).

### 3.6. Publication bias

**Figure 5:**
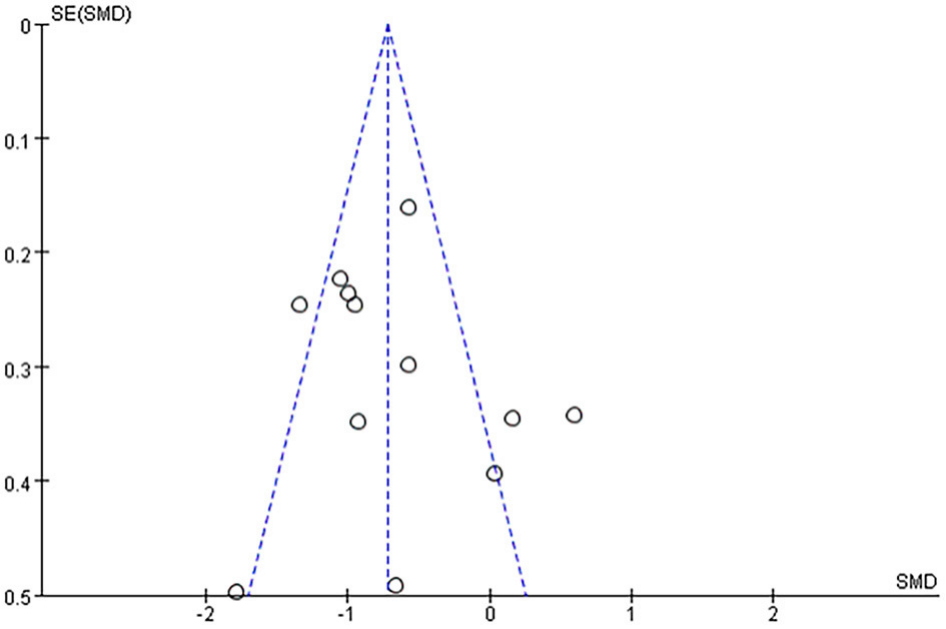
Funnel plot of the included studies on depression.

The publication bias of depression was assessed by visual observation of funnel plots. From the funnel plot distribution, scattered points mostly gathered in the middle and upper parts and moved closer to the middle, but there were also phenomena near the bottom that dispersed outwards. Overall, the results were slightly asymmetrical, suggesting that there may be potential publication bias in the included studies that could influence the results (Figure 5).

## 4. Discussion

### 4.1. Primary outcomes of the review

Reminiscence therapy is a common psychosocial intervention that is recognized as an effective way to improve the health and wellbeing of older adults. Compared with other reviews in this field, our review only focuses on older adults without significant cognitive impairment, including more comprehensive studies and more experimental evidence. Depression and life satisfaction showed significant effects after meta-analysis and subgroup analysis of pooled data.

#### 4.1.1. Depression

The SMD result for depressive symptoms was −0.61 (95% CI: −0.94, −0.28), and the effect size was between medium and large (46). The findings provided some convincing evidence that reminiscence can be beneficial for reducing depression in older adults without obvious cognitive impairment (47, 48). It is worth noting that although older adults are not diagnosed with depression, they usually suffer from depressive symptoms related to long-term stressors and negative emotions (49), so they exhibit low interest and immerse themselves in reflection on negative life experiences. Studies on individual and group reminiscence have both reported improvements in depressive mood. The reason is that reminiscence therapy not only helps to provide pathways for older adults to express unsolved emotions, unknown fears and negative stress to prevent and improve depressed moods (50), but also effectively mobilizes positive emotions by evoking pleasant memories of the past and stimulating conversations with tangible reminders, such as old photos, historical objects, videos and songs (51). However, during reminiscence therapy, recalling negative life events will likely deepen non-pleasant memories and gradually perpetuate depressed mood in older adults (52). Therefore, it was recommended to use structured and positive reminiscence themes to avoid negative memories caused by the free recall to aggravating depression mood.

#### 4.1.2. Life satisfaction

The results showed that the SMD for life satisfaction was 0.40 (95% CI: 0.21, 0.58), indicating that reminiscence therapy significantly improved the life satisfaction of older adults, with an effect size between small and medium (46). Reminiscence plays a positive role in restoring, maintaining or enhancing meaning and satisfaction in later life, transforming negative events into positive outcomes, and is directly related to life satisfaction (53). The self-positive function of recall is especially important when people tend to ignore positive information and indulge in memories that support their dysfunctional thoughts (54). The reminiscence process can help people find more complete and detailed life stories, reinterpret them in a way they understand, and view their lives in a more realistic and objective way (31). In the process of weaving life stories, they will give practical meaning to their past experiences, not just objective judgment and negative reflection (55). Therefore, reminiscence therapy can effectively mobilize the positive emotions of older adults and improve their sense of expectation and goals in life. This is consistent with previous reviews reporting that reminiscence therapy improves life satisfaction in older adults (56). It was worth noting that the factors associated with life satisfaction were different for men and women (57). Therefore, future research could categorize the population and choose more suitable reminiscence types and themes for precise interventions, which might preferably improve the life satisfaction of older adults.

#### 4.1.3. Other psychosocial outcomes

We assessed other psychosocial outcomes, such as self-esteem, loneliness, happiness, and anxiety. Although meta-analyses were not performed due to the limited number of studies related to these outcomes and the large heterogeneity of pooled data, we observed that most studies showed multifaceted benefits of reminiscence therapy as an active intervention for older adults. Reminiscence therapy provides participants with opportunities for interaction and interpersonal communication and enhances a sense of meaning in life (58). During reminiscence and interactive feedback, participants experienced empathetic understanding and self-improvement, which improved their psychological wellbeing (42). Meeting other people with similar psychological problems and life experiences can make older adults realize that they are not alone (59). Reminiscing helps older adults to internally re-evaluate their past while reviewing past experiences, leading to better attitudes and higher levels of happiness (34). In the process of sharing meaningful life experiences, they more actively express themselves and share feelings, thus reducing loneliness and enhancing feelings of self-esteem (15). These beneficial effects can be continuously stimulated and reinforced during the recall process, thus enhancing and sustaining their feeling of happiness, ultimately improving the quality of life among older adults and reducing negative emotions such as anxiety (35). In conclusion, reminiscence therapy plays a positive role in the overall psychosocial wellbeing of older adults (56, 60). Future research should continue to explore the effects of reminiscence therapy on the broader psychological outcomes of older adults, such as adaptation difficulties, perceived stress, and quality of life, to provide more empirical support for the application of reminiscence therapy.

### 4.2. Form of intervention

Based on analyzable outcome indicators, we divided intervention forms into individual reminiscence and group reminiscence for subgroup analysis. Results show that more studies employed group sessions and reported validity on outcomes among older adults without obvious cognitive impairment. Of the seven studies on life satisfaction, six used group sessions, which showed significant statistical significance compared with individual reminiscence (*P* < 0.01). The only study that used individual sessions did not significantly improve life satisfaction (27). In addition, of the twelve studies on depression symptoms, ten used group sessions. And the significant difference was detected between the group and individual sessions (*P* = 0.02). However, while two studies using individual sessions appeared to produce larger effects (SMD: −1.21), seven studies with group sessions all reported significant improvements in depression. This result was consistent with a systematic review by Liu et al. (61) which supported that group reminiscence therapy was more effective than individual reminiscence therapy in reducing depression symptoms in older adults.

Research shows that individual reminiscence therapy is suitable for meeting individual needs, but it requires more manpower, material, time, and resources. The form of individual reminiscence therapy is face-to-face, which cannot provide a broad social platform for older adults. However, group reminiscence therapy focuses more on promoting the integration and interaction between older adults and the environment, helping to establish social networks and obtain a sense of social support and belonging. Taking into account the economic input per capita, group reminiscence therapy is more cost effective than individual therapy (62). Therefore, based on previous evidence and data analyzed, it can be fully demonstrated that the overall benefit of group reminiscence is higher than that of individual reminiscence (63).

### 4.3. Duration of intervention

Previous review studies have realized the potential impact of duration on intervention, but most of them are descriptive analyses in the form of narratives. Based on sufficient analyzable data, we conducted a subgroup analysis of intervention duration, which provided data support for the optimal intervention duration of reminiscence therapy among older adults without obvious cognitive impairment. For the outcome indicator of depression symptoms, intervention durations <8 weeks (3–6 weeks), equal to 8 weeks, and longer than 8 weeks (9–16 weeks) were statistically significant. Therefore, when designing reminiscence therapy for the improvement of depression symptoms in older adults based on resource conservation and optimal effects, the duration of intervention does not need to be too long, but it must be more than 3 weeks. As Stinson et al. (17) found that depression symptoms scores were measured in older adults at 3 and 6 weeks of reminiscence therapy, indicating that the intervention duration had to be more than 3 weeks to observe a significant improvement in depression scores. In addition, only when the duration of reminiscence was more than 8 weeks (9–16 weeks) was the improvement in life satisfaction of older adults statistically significant (*P* < 0.00001). The results illustrated that the same intervention duration had different effects on different psychological outcomes. Thus, when designing the program of reminiscence therapy to simultaneously improve depression and life satisfaction of older adults, interventions should last for more than 8 weeks to ensure that the overall effect is significant. A study found that life satisfaction was affected by many dimensions, including lifestyle and family relationships (64). Therefore, it may take longer to observe an improvement in life satisfaction among older adults, which was consistent with our results (16, 27). However, there is no evidence of a significant correlation between the duration of each intervention and the effectiveness of the outcome. Therefore, the duration of each intervention needs to be reported more to explore the evidence for obtaining the best effect of reminiscence therapy under the best duration of each intervention.

### 4.4. Setting of intervention

The intervention setting of reminiscence therapy was divided into community and care home for subgroup analysis. The results showed that reminiscence therapy had a greater effect on the life satisfaction of older adults living in care homes than on adults in the community. This is in contrast to the findings of Bohlmeijer et al. (60). Data showed that reminiscence therapy significantly improved life satisfaction for care home residents (*P* < 0.00001), but no significant effect was detected for community residents (*P* = 0.51). Coincidentally, both community-implemented studies had an intervention duration of fewer than 8 weeks, which may have contributed to this result. Studies indicate that older adults in care homes are more lacking in attention (65), and they are easy to receive social support and wellbeing through interventions and activities. In addition, different intervention settings had significant differences in depression symptoms of older adults (*p* = 0.02), and the effect in the community was larger (SMD:−1.12). Research suggests that community older adults may have lower negative status than those in care homes and institutions (66), and depression symptoms are worse among older adults in care homes (67). In addition we observed that most of the research data came from care homes, with fewer community-implemented studies. Therefore, reminiscence therapy should be further explored in the community to extend and contrast existing research findings.

## 5. Limitations

This meta-analysis review had some limitations. First, this review pays more attention to published studies in both Chinese and English, which may be a potential bias. Second, this review included as many eligible trials as possible, but the overall quality was limited. On the one hand, we do not deny the possibility that these studies have incomplete data and other biases, and even some studies that do not provide the mean and/or standard deviation are excluded. On the other hand, the quality assessment tool emphasizes blindness and allocation concealment, resulting in a harsh score for reminiscence therapy trials. As a result, the strength of evidence in the present study is subject to certain restrictions. Third, although we evaluated the effects of reminiscence therapy on psychosocial outcomes in older adults without significant cognitive impairment, only very limited research data (e.g., depression and life satisfaction) were available for meta-analysis. When exploring intervention duration and intervention setting on life satisfaction, two studies led to the same results in the same subgroups. Therefore, more research data are needed in the future to explore whether the between-group differences are driven by intervention duration or setting. Fourth, we tried to pay attention to the impact of different follow-up periods on outcome indicators, but the time ranged from 1 day to 6 months. Although we collected 1-month and 3-month follow-up data on depression, the data were limited and the heterogeneity between subgroups was too large to pool. Therefore, future research on reminiscence therapy should focus on the practice of long-term effects.

## 6. Conclusion

In conclusion, reminiscence therapy is a worthwhile intervention for reducing depression symptoms and life satisfaction and improving self-esteem, while reducing loneliness and anxiety in older adults without obvious cognitive impairment. Group reminiscence was used more frequently and the overall effect was better. Different psychological outcomes had different effects with the same intervention duration and setting. Thus, more well-designed trials with large sample sizes and long-term follow-up are necessary to confirm and expand the present results.

## Data availability statement

The original contributions presented in the study are included in the article/Supplementary material, further inquiries can be directed to the corresponding author.

## Author contributions

Study conception and design: LX and SL. Data collection and evaluation: YN, YW, and YL. Data extraction and analysis: LX, SL, and RY. Manuscript draft: LX, SL, RY, YN, YW, and YL. Critical revision of important intellectual content: SL and RF. All authors contributed to the article and approved the submitted version.

## References

[1] United Nations Department Department of Economic and Social Affairs Population Division. World Population Prospects 2019: Highlights. (2019). Available online at: https://population.un.org/wpp/Publications/Files/WPP2019_Highlights.pdf (accessed October 1, 2022).

[2] Rudnicka E Napierała P Podfigurna A Meczekalski B Smolarczyk R Grymowicz M . The World Health Organization (WHO) approach to healthy ageing. Maturitas. (2020) 139:6–11. 10.1016/j.maturitas.05,018. 32747042 PMC7250103

[3] Sánchez-González D Rojo-Pérez F Rodríguez-Rodríguez V Fernández-Mayoralas G. Environmental and psychosocial interventions in age-friendly communities and active ageing: a systematic review. Int J Environ Res Public Health. (2020) 17:8305. 10.3390/ijerph17228305 33182710 PMC7696667

[4] Nelson CJ Saracino RM Roth AJ Harvey E Martin A Moore M . Cancer and aging: reflections for elders (CARE): a pilot randomized controlled trial of a psychotherapy intervention for older adults with cancer. Psychooncology. (2019) 28:39–47. 10.1002/pon.4907 30296337 PMC6476184

[5] Li A Liu Y. Reminiscence therapy serves as an optional nursing care strategy in attenuating cognitive impairment, anxiety, and depression in acute ischemic stroke patients. Ir J Med Sci. (2022) 191:877–84. 10.1007/s11845-021-02600-8 33755917

[6] Westerhof GJ Bohlmeijer E Webster JD. Reminiscence and mental health: a review of recent progress in theory, research and interventions. Ageing Soc. (2010) 30:697–721. 10.1017/S0144686X09990328

[7] Syed Elias SM Neville C Scott T. The effectiveness of group reminiscence therapy for loneliness, anxiety and depression in older adults in long-term care: a systematic review. Geriatr Nurs. (2015) 36:372–80. 10.1016/j.gerinurse.05004 26099638

[8] Shellman JM Mokel M Hewitt N. The effects of integrative reminiscence on depressive symptoms in older African Americans. West J Nurs Res. (2009) 31:772–86. 10.1177/0193945909335863 19448051

[9] Meléndez-Moral JC Fortuna FB Sales A Mayordomo T. The effects of instrumental reminiscence on resilience and coping in elderly. Arch Gerontol Geriatr. (2014) 60:294–8. 10.1016/j.archger.12,001. 25555754

[10] Roback HB. Adverse outcomes in group psychotherapy: risk factors, prevention, and research directions. J Psychother Pract Res. (2000) 9:113–22. 10896735 PMC3330596

[11] Wu D Chen T Yang H Gong Q Hu X. Verbal responses, depressive symptoms, reminiscence functions and cognitive emotion regulation in older women receiving individual reminiscence therapy. J Clin Nurs. (2018) 27:2609–19. 10.1111/jocn.14156 29119637

[12] Amieva H Robert PH Grandoulier AS Meillon C De Rotrou J Andrieu S . Group and individual cognitive therapies in Alzheimer's disease: the ETNA3 randomized trial. Int Psychogeriatr. (2016) 28, 707–717. 10.1017/s1041610215001830 26572551

[13] Bai Z Shen J. Effect of individual reminiscence therapy and group reminiscence therapy on the depression of elderly people in pension institutions. J Chongqing Med Univ. (2018) 43:1083–9. 10.13406/j.cnki.cyxb.001743

[14] Woods B O'Philbin L Farrell EM Spector AE Orrell M. Reminiscence therapy for dementia. Cochrane Database Syst Rev. (2018) 3:Cd001120. 10.1002/14651858.CD001120.pub3 29493789 PMC6494367

[15] Chao SY Liu HY Wu CY Jin SF Chu TL Huang TS . The effects of group reminiscence therapy on depression, self esteem, and life satisfaction of elderly nursing home residents. J Nurs Res. (2006) 14:36–45. 10.1097/01.jnr.0000387560.03823.c7 16547904

[16] Choy JC Lou VW. Effectiveness of the modified instrumental reminiscence intervention on psychological well-being among community-dwelling chinese older adults: a randomized controlled trial. Am J Geriatr Psychiatry. (2015) 24:60–9. 10.1016/j.jagp.05008 26419735

[17] Stinson CK Young EA Kirk E Walker R. Use of a structured reminiscence protocol to decrease depression in older women. J Psychiatr Ment Health Nurs. (2010) 17:665–73. 10.1111/j.1365-201001556.x 21050332

[18] Aşiret GD Dutkun M. The effect of reminiscence therapy on the adaptation of elderly women to old age: a randomized clinical trial. Complement Ther Med. (2018) 41:124–9. 10.1016/j.ctim.09018 30477828

[19] Aşiret GD. Effect of reminiscence therapy on the sleep quality of the elderly living in nursing homes: a randomized clinical trial. Eur J Integr Med. (2018) 20:1–5. 10.1016/j.eujim.03007

[20] Mei Y Lin B Li Y Ding C Zhang Z. Effects of modified 8-week reminiscence therapy on the older spouse caregivers of stroke survivors in Chinese communities: a randomized controlled trial. Int J Geriatr Psychiatry. (2018) 33:633–41. 10.1002/gps.4833 29266450

[21] Tam W Poon SN Mahendran R Kua EH Wu XV. The effectiveness of reminiscence-based intervention on improving psychological well-being in cognitively intact older adults: a systematic review and meta-analysis. Int J Nurs Stud. (2021) 114:103847. 10.1016/j.ijnurstu.2020.103847 33352435

[22] Lan X Xiao H Chen Y. Effects of life review interventions on psychosocial outcomes among older adults: a systematic review and meta-analysis. Geriatr Gerontol Int. (2017) 17:1344–57. 10.1111/ggi.12947 28124828

[23] Higgins JPT Deeks JJ Altman DG. Cochrane Handbook for Systematic Reviews of Interventions Version 5, 1.0 [updated March 2011]. (2011). The Cochrane Collaboration. Available online at: https://www.cochrane-handbook.org

[24] Thomas BH Ciliska D Dobbins M Micucci S. A process for systematically reviewing the literature: providing the research evidence for public health nursing interventions. Worldviews Evid Based Nurs. (2004) 1:176–84. 10.1111/j.1524-475X.2004.04006.x 17163895

[25] Armijo-Olivo S Stiles CR Hagen NA Biondo PD Cummings GG. Assessment of study quality for systematic reviews: a comparison of the cochrane collaboration risk of bias tool and the effective public health practice project quality assessment tool: methodological research. J Eval Clin Pract. (2012) 18:12–8. 10.1111/j.1365-2010,01516.x 20698919

[26] Cook EA. The effects of reminiscence on psychological measures of ego integrity in elderly nursing home residents. Arch Psychiatr Nurs. (1991) 5:292–8. 10.1016/0883-9417(91)90027-3 1750779

[27] Ligon MB. Improving Life Satisfaction of Elders through Oral History: The Narrator's Perspective [dissertation/master's thesis]. Virginia Commonwealth University (2007).

[28] Norris T. The Effectiveness and Perceived Effectiveness of Simple Reminiscence Therapy Involving Photographic Prompts for Determining Life Satisfaction in Non-institutionalized Elderly Persons. [dissertation/master's thesis]. Louisiana State University Health Sciences Center School of Nursing. (2001).

[29] Sabir M Henderson CR Kang SY Pillemer K. Attachment-focused integrative reminiscence with older African Americans: a randomized controlled intervention study. Aging Ment Health. (2016) 20:517–28. 10.1080/13607863.2015.1023764 25812080 PMC4583805

[30] Kaplan T Keser I. The effect of individual reminiscence therapy on adaptation difficulties of the elderly: a randomized clinical trial. Psychogeriatrics. (2021) 21:869–80. 10.1111/psyg.12761 34530495

[31] Viguer P Satorres E Fortuna FB Meléndez JC. (2017). A follow-up study of a reminiscence intervention and its effects on depressed mood, life satisfaction, and well-being in the elderly. J Psychol. (23980) 151:789–803. 10.1080/00220171393379 29166223

[32] Satorres E Viguer P Fortuna FB Meléndez JC. Effectiveness of instrumental reminiscence intervention on improving coping in healthy older adults. Stress Health. (2018) 34:227–34. 10.1002/smi.2776 28834143

[33] Pishvaei M Ataie Moghanloo R Ataie Moghanloo V. The efficacy of treatment reminders of life with emphasis on integrative reminiscence on self-esteem and anxiety in widowed old men. Iran J Psychiatry. (2015) 10:19–24. 26005476 PMC4434424

[34] Yousefi Z Sharifi K Tagharrobi Z Akbari H. The effect of narrative reminiscence on happiness of elderly women. Iran Red Crescent Med J. (2015) 17:e19612. 10.5812/ircmj.19612 26734470 PMC4698128

[35] Bazrafshan MR Jokar M Soufi O Delam H. The effect of structured group reminiscence on depression and anxiety of the elderly female hookah users. J Subst Use. (2021) 27:528–34. 10.1080/14659891.2021.1967479

[36] Meléndez-Moral JC Charco-Ruiz L Mayordomo-Rodríguez T Sales-Galán A. Effects of a reminiscence program among institutionalized elderly adults. Psicothema. (2013) 25:319–23. 10.7334/psicothema2012.253 23910745

[37] Satorres E Delhom I Meléndez JC. Effects of a simple reminiscence intervention program on the reminiscence functions in older adults. Int Psychogeriatr. (2021) 33:557–66. 10.1017/s1041610220000174 32063238

[38] Syed Elias SM Neville C Scott T Petriwskyj A. (2020). The effectiveness of spiritual reminiscence therapy for older people with loneliness, anxiety and depression in Malaysia. J Relig Spiritual Aging. (28030) 32:341–56. 10.1080/15520201765448

[39] Saredakis D Keage HA Corlis M Ghezzi ES Loffler H Loetscher T . The effect of reminiscence therapy using virtual reality on apathy in residential aged care: multisite nonrandomized controlled trial. J Med Internet Res. (2021) 23:e29210. 10.2196/29210 34542418 PMC8491119

[40] Sok SR. Effects of individual reminiscence therapy for older women living alone. Int Nurs Rev. (2015) 62:517–24. 10.1111/inr.12190 25891307

[41] Wu LF. Group integrative reminiscence therapy on self-esteem, life satisfaction and depressive symptoms in institutionalised older veterans. J Clin Nurs. (2001) 20:2195–203. 10.1111/j.1365-201103699.x 21631615

[42] Chiang KJ Chu H Chang HJ Chung MH Chen CH Chiou HY . The effects of reminiscence therapy on psychological well-being, depression, and loneliness among the institutionalized aged. Int J Geriatr Psychiatry. (2010) 25:380–8. 10.1002/gps.2350 19697299

[43] Li S Dai Y Zhou Y Zhang J Zhou C. Efficacy of group reminiscence therapy based on Chinese traditional festival activities (CTFA-GRT) on loneliness and perceived stress of rural older adults living alone in China: a randomized controlled trial. Aging Ment Health. (2021) 26:1377–84. 10.1080/13607863.2021.1935457 34180278

[44] Wang X Zhang JF Li ZJ. Influence of reminiscence therapy on depression and happiness degree of empty nest elderly in community. Chin Nurs Res. (2011) 25:3192–4. 10.3892/ijmm.2011.859 22159350

[45] Liu SJ Lin CJ Chen YM Huang XY. The effects of reminiscence group therapy on self-esteem, depression, loneliness and life satisfaction of elderly people living alone. Mid-Taiwan Journal of Medicine. (2007) 12:133–42.

[46] Cohen J. Statistical Power Analysis for the Behavioral Sciences. Hillsdale, NJ: Lawrence Erlbaum Associates (1988).

[47] Bohlmeijer E Smit F Cuijpers P. Effects of reminiscence and life review on late-life depression: a meta-analysis. Int J Geriatr Psychiatry. (2003) 18:1088–94. 10.1002/gps.1018 14677140

[48] Hsieh HF Wang JJ. Effect of reminiscence therapy on depression in older adults: a systematic review. Int J Nurs Stud. (2003) 40:335–45. 10.1016/s0020-7489(02)00101-3 12667510

[49] Blazer DG. Depression in late life: review and commentary. J Gerontol A Biol Sci Med Sci. (2003) 58:249–65. 10.1093/gerona/58.3.m249 12634292

[50] Fry PS. Structured and unstructured reminiscence training and depression among the elderly. Clin Gerontol. (1983) 1:15–37. 10.1300/J018v01n03_0619904866

[51] Spector AE Orrell M Davies SP Woods RT. Reminiscence therapy for dementia. Cochrane Database Syst Rev. (2000) 5:85–6. 10.1002/14651858.CD001120 11034700

[52] Connolly SL Alloy LB. Negative event recall as a vulnerability for depression: relationship between momentary stress-reactive rumination and memory for daily life stress. Clin Psychol Sci. (2018) 6:32–47. 10.1177/2167702617729487 29552424 PMC5849261

[53] Cappeliez P O'Rourke N. Empirical validation of a model of reminiscence and health in later life. J Gerontol. (2006) 61:237–44. 10.1093/geronb/61.4.P237 16855036

[54] O'Rourke N Cappeliez P Claxton A. Functions of reminiscence and the psychological well-being of young-old and older adults over time. Aging Ment Health. (2011) 15:272–81. 10.1080/13607861003713281 21140308

[55] Watt LM Cappeliez P. Integrative and instrumental reminiscence therapies for depression in older adults: intervention strategies and treatment effectiveness. Aging Ment Health. (2000) 4:166–77. 10.1080/1360786005000869120737322

[56] Lin YC Dai YT Hwang SL. The effect of reminiscence on the elderly population: a systematic review. Public Health Nurs. (2013) 20:297–306. 10.1046/j.1525-200320407.x 12823790

[57] Berg AI Hassing LB McClearn GE Johansson B. What matters for life satisfaction in the oldest-old? Aging Mental Health. (2006) 10:257–64. 10.1080/13607860500409435 16777653

[58] Gaggioli A Scaratti C Morganti L Stramba-Badiale M Agostoni M Spatola CA . Effectiveness of group reminiscence for improving wellbeing of institutionalized elderly adults: study protocol for a randomized controlled trial. Trials. (2014) 15:408. 10.1186/1745-6215-15-408 25344703 PMC4216871

[59] Allen AP Doyle C Roche R. The impact of reminiscence on autobiographical memory, cognition and psychological well-being in healthy older adults. Eur J Psychol. (2020) 16:317–30. 10.5964/ejop.v16i2.2097 33680185 PMC7913011

[60] Bohlmeijer E Roemer M Cuijpers P Smit F. The effects of reminiscence on psychological well-being in older adults: a meta-analysis. Aging Ment Health. (2007) 11:291–300. 10.1080/13607860600963547 17558580

[61] Liu Z Yang F Lou Y Zhou W Tong F. The effectiveness of reminiscence therapy on alleviating depressive symptoms in older adults: a systematic review. Front Psychol. (2021) 12:709853. 10.3389/fpsyg.2021.709853 34484066 PMC8415872

[62] Fakhar F Navabinejad S Foroghan M. The effect of group counseling with therapeutic approach on the mental health of female elderly. Salmand. (2008) 3:58–65.

[63] Tavares LR Barbosa MR. Efficacy of group psychotherapy for geriatric depression: a systematic review. Arch Gerontol Geriatr. (2018) 78:71–80. 10.1016/j.archger.06001 29933137

[64] Fastame MC. Life satisfaction in late adult span: the contribution of family relationships, health self-perception and physical activity. Aging Clin Exp Res. (2021) 33:1693–8. 10.1007/s40520-020-01658-1 32700295

[65] Shah R Carandang RR Shibanuma A Ong KIC Kiriya J Jimba M . Understanding frailty among older people living in old age homes and the community in Nepal: a cross-sectional study. PLoS One. (2021) 16:e0251016. 10.1371/journal.pone.0251016 33914828 PMC8084172

[66] Mitchell UA Ailshire JA Brown LL Levine ME Crimmins EM. Education and Psychosocial Functioning Among Older Adults: 4-Year Change in Sense of Control and Hopelessness. J Gerontol B Psychol Sci Soc Sci. (2018) 73:849–59. 10.1093/geronb/gbw031 27013537 PMC6283311

[67] Finnegan S Bruce J Lamb SE Griffiths F. Predictors of attendance to group exercise: a cohort study of older adults in long-term care facilities. BMC Geriatr. (2015) 15:37. 10.1186/s12877-015-0043-y 25887989 PMC4392629

