# Supplementary material for: Effects of reminiscence therapy on psychological outcome among older adults without obvious cognitive impairment: A systematic review and meta-analysis
Source: Front Psychiatry. 2023 Mar 30;14:1139700. doi: 10.3389/fpsyt.2023.1139700 (PMC10098219; doi:10.3389/fpsyt.2023.1139700)

**Supplementary Figure1**. Forest plot results for self-esteem, loneliness, anxiety, and happiness.

Outcome: self-esteem

| | Experimental | | | Control | | | | Std. Mean Difference |
|---|---|---|---|---|---|---|---|---|
| Study or Subgroup | Mean | SD | Total | Mean | SD | Total | Weight | IV, Random, 95% CI |
| Chao 2006 | 29.18 | 4 | 10 | 25.88 | 1.96 | 8 | 19.4% | 0.96 [-0.03, 1.96] |
| Cook 1991 | 1.42 | 1 | 18 | 1.85 | 0.75 | 18 | 21.0% | -0.48 [-1.14, 0.19] |
| Liu 2007 | 24.4 | 3.1 | 12 | 21.2 | 4.2 | 14 | 20.4% | 0.83 [0.02, 1.64] |
| Pishvaei 2015 | 22.53 | 3.86 | 17 | 9.59 | 0.87 | 17 | 17.6% | 4.52 [3.19, 5.84] |
| Wu 2010 | 26.71 | 2.67 | 35 | 23.9 | 1.67 | 39 | 21.6% | 1.26 [0.76, 1.77] |
| **Total (95% CI)** | | | **92** | | | **96** | **100.0%** | **1.32 [0.12, 2.53]** |

Heterogeneity: Tau² = 1.67; Chi² = 47.14, df = 4 (P < 0.00001); I² = 92%
Test for overall effect: Z = 2.16 (P = 0.03)

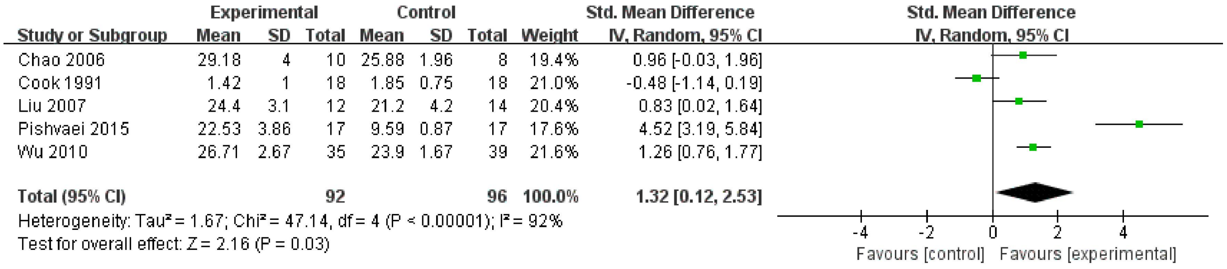


Outcome: loneliness

| | Experimental | | | Control | | | | Std. Mean Difference |
|---|---|---|---|---|---|---|---|---|
| Study or Subgroup | Mean | SD | Total | Mean | SD | Total | Weight | IV, Random, 95% CI |
| Chiang 2010 | 34.82 | 7.05 | 45 | 42.11 | 7.82 | 47 | 26.2% | -0.97 [-1.40, -0.54] |
| Li 2021 | 47.43 | 1.12 | 30 | 57.47 | 1.13 | 30 | 22.4% | -8.81 [-10.52, -7.10] |
| Liu 2007 | 35.5 | 3.4 | 12 | 35.8 | 7 | 14 | 25.6% | -0.05 [-0.82, 0.72] |
| Syed Elias 2020 | 41.89 | 10.95 | 18 | 41.5 | 5.45 | 16 | 25.8% | 0.04 [-0.63, 0.72] |
| **Total (95% CI)** | | | **105** | | | **107** | **100.0%** | **-2.23 [-4.28, -0.18]** |

Heterogeneity: Tau² = 4.13; Chi² = 94.65, df = 3 (P < 0.00001); I² = 97%
Test for overall effect: Z = 2.13 (P = 0.03)

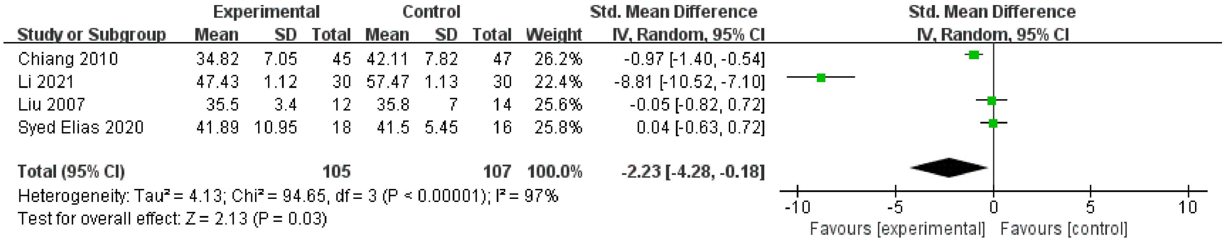


Outcome: anxiety

| | Experimental | | | Control | | | | Std. Mean Difference |
|---|---|---|---|---|---|---|---|---|
| Study or Subgroup | Mean | SD | Total | Mean | SD | Total | Weight | IV, Random, 95% CI |
| Bazrafshan 2021 | 42.5 | 6.2 | 12 | 48.58 | 6.29 | 12 | 33.7% | -0.94 [-1.79, -0.09] |
| Pishvaei 2015 | 24.71 | 3.86 | 17 | 45.29 | 5.8 | 17 | 31.9% | -4.08 [-5.31, -2.85] |
| Syed Elias 2020 | 23.89 | 12.98 | 18 | 20.62 | 12.18 | 16 | 34.3% | 0.25 [-0.42, 0.93] |
| **Total (95% CI)** | | | **47** | | | **45** | **100.0%** | **-1.53 [-3.73, 0.67]** |

Heterogeneity: Tau² = 3.55; Chi² = 36.67, df = 2 (P < 0.00001); I² = 95%
Test for overall effect: Z = 1.37 (P = 0.17)

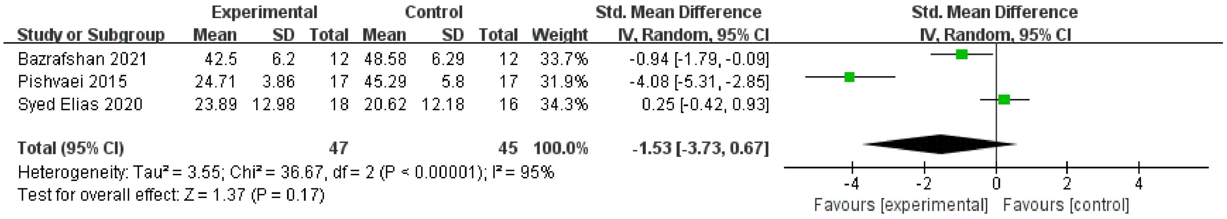


Outcome: happiness

| | Experimental | | | Control | | | | Std. Mean Difference |
|---|---|---|---|---|---|---|---|---|
| Study or Subgroup | Mean | SD | Total | Mean | SD | Total | Weight | IV, Random, 95% CI |
| wang 2011 | 32.69 | 6.95 | 42 | 22.8 | 6.32 | 40 | 53.1% | 1.47 [0.98, 1.96] |
| Yousefi 2015 | 46.93 | 8.093 | 15 | 43.5 | 10.603 | 14 | 46.9% | 0.36 [-0.38, 1.09] |
| **Total (95% CI)** | | | **57** | | | **54** | **100.0%** | **0.95 [-0.14, 2.04]** |

Heterogeneity: Tau² = 0.52; Chi² = 6.15, df = 1 (P = 0.01); I² = 84%
Test for overall effect: Z = 1.70 (P = 0.09)

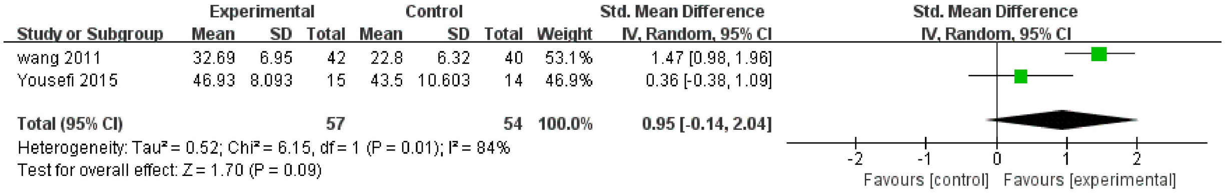

Supplement: Supplementary file 1 [file Data_Sheet_1.ZIP › supplementary materials/Supplementary Figure1.pdf]
