# Supplementary material for: Effects of reminiscence therapy on psychological outcome among older adults without obvious cognitive impairment: A systematic review and meta-analysis
Source: Front Psychiatry. 2023 Mar 30;14:1139700. doi: 10.3389/fpsyt.2023.1139700 (PMC10098219; doi:10.3389/fpsyt.2023.1139700)
Supplement: Supplementary file 1 [file Data_Sheet_1.ZIP › supplementary materials/Supplementary Table 1.pdf]

Supplementary Table 1. Characteristics of the included studies.

Author (Year)	Country; Setting	Mean Age (SD) Female(%)	Analyzed (n)/Post-test I/C	Intervention; Type & Form	Topics included in reminiscence therapy	Design, Duration & Frequency, Follow-up	Control & Instructor	Outcome (measures)
Aşiret (2018)	Turkey; Nursing home	76.02 (SD = 7.31) (41.3%)	46 22/24	Non-specified reminiscence therapy; Individual	Topics for 12 weeks included: (1) Self-introduction; (2) Childhood and family life; (3) School days; (4) Starting work and work life; (5) A fun day out of the home; (6) Marriage (friendship for the singles); (7) Plants and animals they loved; (8) Babies and children; (9) Food and cooking; (10) Holidays and travelling; (11) Special days and celebrations; (12) Assessment and closure.	RCT 12 sessions, 25-30 min; weekly; Not mentioned	No intervention; Researcher	1. Sleep quality (PSQI)
Aşiret & Dutkun (2018)	Turkey; Family health centre	72.24 (SD = 6.29) (100%)	50 27/23	Non-specified reminiscence therapy; Individual	Topics for 8 weeks included：(1) Introduction and beginning; (2) Childhood and family life; (3) Marriage and children (friendship for single participation); (4) Food and cooking; (5) Religious and official holidays; (6) An entertaining day out of home; (7) Vacation and travelling; (8) Assessment and closing.	RCT 8 sessions, 30-45 min; weekly; Weekly home visit	No intervention; Researcher	1. Cognitive status (MMSE) 2. Adaptation to old age (ASADE)
Bazrafshan (2021)	Iranian；Health centers	⩾ 60 (Mean age not reported) (50%)	24 12/12	Structured reminiscence therapy; Group	Topics for 6 weeks included: (1) Introduction; Childhood and family life; (2) School-age; a career and a professional life; (3) Going to the open space area; Marriage; (4) Home, garden and favorite animals; Next generation, infancy, and childhood period; (5) Foods, cooking; Vacation, journeys; (6) Celebration; Summarize.	RCT 12 sessions, 1-1.5 hr each; weekly; 1-month follow-up	No intervention; Researchers	1. Depression (GDS-15) 2. Anxiety (STAI)
Chao (2006)	Taiwan, China; Nursing home	65 to 85 (Mean age not reported) (25%)	24 12/12	Non-specified reminiscence therapy; Group	Topics for 9 weeks included: (examples only provided) Traditional Chinese holidays; History of childhood/family; Significant memories in their life.	Quasi-experimental Study 9 sessions, 1 hr each; weekly; Not mentioned	No intervention; A qualified instructor and a head nurse.	1. Depression (GDS-S) 2. Self-esteem (RSES) 3. Life satisfaction (QLI)
Chiang (2010)	Taiwan, China; Nursing home	77.24 (SD=3.97) (0%)	92 45/47	Non-specified reminiscence therapy; Group	Topics for 8 weeks included: (1) Sharing memories and greeting; (2) Expressing their feelings; (3) Positive relationships; (4) Family history and life stories; (5) Transition in life issues; (6) Personal accomplishments and goals; (7) Positive strengths and goals; (8) Review and summary.	RCT 8 sessions, 1.5 hr each; weekly; 3-month follow-up	Waiting List; A master's prepared student in mental health nursing	1. Depression mood (CES-D) 2. Psychological well-being (SCL-90-R) 3. Loneliness (RULS-V3)
Choy & Lou (2016)	Hong Kong, China; Empty nest community	⩾ 60 (Mean age not reported) (64.9%)	81 39/42	Instrumental reminiscence therapy; Group	Contents for 6 weeks included: (examples only provided) Branching points; family; Major life events; Career. Sequential components: Introducing and reviewing homework, Relaxation and refocusing exercise, Discussion theme and contact work, Feedback from group members, Discussion of homework and questions and feedback.	RCT 6 sessions,1.5 hr each; weekly; 2-week & 4-week follow-up	Waiting List; Led by a registered social worker and a training observer	1. Depression (GDS-15) 2. Life satisfaction (LSS-C)

Supplementary Table 1. (Continued)

Cook (1991)	USA; Nursing home	81.30 (65 to 96) (87.8%)	54 18/18/18	Non-specified reminiscence therapy; Group	Topics for 16 weeks included: (examples only provided) (1) Reminiscence group: Childhood experiences; Marriage; Family life and jobs every decade. (2) Current events group: Thinking and talking about the present and the future.	RCT 16 sessions, 1 hr each; weekly; Not mentioned	1.Current events 2.No-treatment Nurse	1. Life satisfaction (LSI-A) 2. Self-esteem (RSES) 3. Depression (GDS-30)
Kaplan & Keser (2021)	Turkey; Family health centre	⩾ 65 (Mean age not reported) (47.5%)	65 31/34	Non-specified reminiscence therapy; Individual	Topics for 8 weeks included: (Non-sequential) (1) Childhood; (2) Business life; (3) Married life; (4) Old items; (5) Holidays; (6) Traditional dishes; (7) Songs; (8) Movies.	RCT 8 sessions, 30-45min; weekly; 2-month follow-up	No intervention; A psychiatric nursing graduate student	1. Adaptation difficulty (ASADE) 2. Nurses’ observation (NOSGER)
Li (2021)	China; Rural	65.70 (SD =3.69) (63.3%)	60 30/30	Non-specified reminiscence therapy; Group	Topics for 8 months included: (1) Kitchen god Festival: family; (2) Lantern Festival: hobbies; (3) International Women’s Day: happy holidays; (4) Tomb Sweeping Day: colourful life; (5) Mother’s Day: love; (6) Dragon Boat Festival: hand in hand; (7) Seventy-one Party Building Festival: go all the way; (8) Chinese Valentine’s Day: new life new self.	RCT 8 sessions, 4 hr each; monthly; 3-month follow-up	No intervention; Professional psychology teachers, nursing graduate students and expert team	1. Perceived stress (PSS) 2. Loneliness (UCLA-LS)
Ligon (2007)	USA; Community	81.10 (SD = 7.07) (75%)	60 30/30	Non-specified reminiscence therapy; Individual	Topics for 3 weeks included: (1) Childhood and youth; (2) Young and middle adulthood; (3) Late life.	Quasi-experimental Study,3 sessions, 1 hr each; weekly; 10-week follow-up	No-treatment; College students enrolled in a gerontology course	1. Life satisfaction (LSI-A)
Liu (2007)	Taiwan, China; Nursing home	74.7 (SD=5.4) (12%)	28 12/14	Non-specified reminiscence therapy; Group	Topics for 10 weeks included: (1) Reminiscence introduction and greeting; (2) Old songs and their background; (3) photographs; (4) Proudest events; (5) Unforgettable love affairs; (6) Most memorable moments; (7) Experiences they most want to share; (8)-(10) Linking their experiences with the present and the future.	RCT 10 sessions, 1 hr each; weekly; Not mentioned	Regular group activities; Principle investigator, three graduate students in nursing and one research assistant	1. Self-esteem (SES) 2. Depression (CES-D) 3. Loneliness (UCLA) 4. Life satisfaction (LSI-A)
Meléndez-Moral (2013)	Spain; Retirement home	79.78 (SD = 9.34) (83.3%)	34	Non-specified reminiscence therapy; Group	Topics for 8 weeks included: (1) From childhood through old age; (2) My town/city; (3) Games from childhood and youth; (4) Popular songs; (5) Holidays and special days; (6) The movies over time; (7)-(8) Remembering my grandmother.	Quasi-experimental Study 8 sessions, 1 hr each; weekly; Not mentioned	Normal activities; Psychologist	1. Depressive symptom (Mini-GDS 8) 2. Life satisfaction (PGCMS) 3. Self-esteem (RSES) 4. Psychological well-being (SPWB)
Meléndez-Moral (2015)	Dominican Republic; Community	73.10 (Mean age not reported) (55.3%)	30 15/15	Instrumental reminiscence therapy; Group	Topics for 8 weeks included: (Simple summary) (1) Inform the concept of reminiscence and the procedure; (2) Elicited memories of everyday places and past things; (3) Interpersonal relationships; (4) Important events and dates; (5) Holidays and traditions; (6) Reflection on escapism; (7) Occupational activities; (8) Main achievements and goals.	RCT 8 sessions,1 hr each; weekly; Not mentioned	Waiting list; Psychologist	1. Resilience (BRCS) 2. Coping (BRCS)

Supplementary Table 1. (Continued)

Norris (2001)	USA; Community	78.64 (SD = 8.73) (70%)	78 25/25/23	Simple reminiscence therapy; Individual	Contents for 4 weeks included: (1) Reminiscence group: Shared personal photographs; Talked about the past with photos. (2) Current events group: Discussed articles in local newspapers.	RCT 4 sessions, 30-40 min; weekly; Not mentioned	1.Current events 2. No-treatment Researcher	1. Life satisfaction (SALSS)
Pishvaei (2015)	Iran; Community	69.71 (60 to 80) (0%)	34 17/17	Integrative Reminiscence therapy; Group	Topics for 8 weeks included: (1) Major turning points in life; (2) Family history; (3) Career; (4) History of loves and hates; (5) Stressful experiences; (6) Meaning and purpose of life.	RCT 6 sessions,1 hr each; weekly; Not mentioned	Normal activities; Psychologist	1. Anxiety (General Anxiety questionnaire) 2. Self-esteem (RSES)
Sabir (2016)	USA; Senior center	72.00 (SD = 8.00) (90%)	62 32/29	Integrative reminiscence therapy; Group	Contents for 8 weeks included: (examples only provided) Got to know each other; Shared a meal together; Shared their historical about their first names.	RCT 8 sessions, 2 hrs each; weekly; 6-month follow-up	Participated the first and last reminiscence activities; A narrative psychologist who specializes in AFIR.	1. Depression (CES-D) 2. Perceived stress (PSS) 3. ER visits 4. Self-rated health 5. Sense of coherence (SOC) 6. Self-efficacy (GSE) 7. Self-acceptance 8. Generativity (LGS)
Satorres et al. (2018)	Dominican Republic; Community	72.11 (SD = 6.23) (54.7%)	150 77/73	Instrumental reminiscence therapy; Group	Contents for 10 weeks included: (examples only provided) Recall past events; Actively participated in the environment; Shared pleasant feelings and past events; Shared the experience of frustration.	RCT 10 sessions, 2 hrs each; weekly; 3-month follow-up	No-treatment; Psychologist	1. Coping strategies (CAE)
Saredakis et al. (2021)	South Australia；Residential aged care facilities	84.8 (SD = 8.0) (65%)	43 15/14/14	Non-specified reminiscence therapy; Individual	Contents for 2 weeks included: (1) Reminiscence group: Using VR to view the content related to the participants' background obtained from the recall interview. (2) Active control group: Viewed the reminiscence content on a laptop computer.	Quasi-experimental Study, 3 sessions, 20 mins each; 1-day & 4-week follow-up	Intervention using laptop computer Usual care Researchers	1. Apathy (AES) 2. Cognitive ability(ACE-III) 3. Depression (GDS)
Satorres et al. (2021)	Spain；Healthcare center	≥ 65 (Mean age was reported) (53.2%)	139 71/68	Simple reminiscence therapy; Group	Topics for 10 weeks included: (1) Reminiscence introduction; (2) Remember past events; (3) Social and family; (4) Pleasant feelings about past events; (5) Narrative traditions; (6) Social and technological changes; (7) Work and occupations; (8) Positive elements of memories; (9) Typical objects from the past; (10) Songs and images.	RCT 10 sessions, 2 hr each; weekly; 3-month follow-up	No intervention; Psychologist	1. Reminiscence functions (RFS)
Shellman (2009)	USA; Community	72.60 (SD = 8.60) (77%)	56 19/19/18	Integrative reminiscence therapy; Individual	Contents for 8 weeks included: (examples only provided) (1) Reminiscence group: Life experience of youth; Family, parents, and siblings; Religion and faith; Special aspirations or dreams of youth; Favorite memory. (2) Health education group: Medication management; Stress management; Prevention of falls; Exercise.	RCT 8 sessions, 45 mins each; weekly; 1-month follow-up	Health education No intervention A research assistant or the PI	1. Depressive symptoms (CES-D)

Supplementary Table 1. (Continued)

Sok (2015)	Korea; Community	⩾ 65 (Mean age not reported) (100%)	78 40/38	Non-specified reminiscence therapy; Individual	Topics for 4 weeks included: (1) Greeting and introducing; Brief introduction of reminiscence therapy; Initial reminiscence therapy; (2) Childhood and adolescence; (3) Adulthood; (4) Senescence and conclusion.	Quasi-experimental Study 4 sessions, 1 hr each; weekly; Not mentioned	Greeting with blood pressure; Researcher	1. Memory self-efficacy (MSEQ) 2. Memory practice (The Memory Practice Method) 3. Cognitive function (MMSE-K) 4. Quality of life (SF-36 QOL)
Stinson (2010)	USA; Assisted living facilities	82.53 (SD = 7.58) (100%)	47 22/25	Structured reminiscence therapy; Group	Topics for 6 weeks included: (examples only provided) (1) Introduction of leaders and members; Remembering the past through songs from the 1920s to 1960s; (2) Shared photographs; Discussing home/work life or volunteer activities/first job.	RCT 12 sessions, 1 hr each; twice a week; Not mentioned	Usual care; Researcher	1. Depression (GDS-30)
Syed Elias (2020)	Malaysia；Residential aged care facility	⩾ 60 (Mean age not reported) (52.9%)	34 18/16	Spiritual reminiscence therapy; Group	Topics for 6 weeks included: (1) Life meaning; (2) Relationships-isolation, connecting; (3) Hopes, fears and worries; (4) Growing older and transcendence; (5) Spiritual and religious belief; (6) Spiritual and religious practices.	RCT 6 sessions, 1-1.5 hr each; weekly; 3-month follow-up	Attention control activities; Researcher	1. Loneliness (UCLA) 2. Anxiety (GAS) 3. Depression (M-GDS-14)
Viguer (2017)	Dominican Republic; Healthcare center	72.25 (SD = 6.70) (54%)	168 84/84	Non-specified reminiscence therapy; Group	Topics for 10 weeks included: (1) Reminiscence introduction; (2) Daily things; (3) Interpersonal relationships; (4) Important dates; (5) Popular holidays; (6) Cinema and advertising; (7) Work and tasks; (8) Traditional games; (9) Remembering their grandmother; (10) Music therapy.	RCT 10 sessions, 2 hrs each; weekly; 3-month follow-up	Waiting list; Psychologist	1. Depressed mood (GDS-30) 2. Life satisfaction (LSI-A) 3. Psychological well-being (PWBS)
Wang (2011)	China; Community	⩾ 60 (Mean age not reported) (57.3%)	82 42/40	Non-specified reminiscence therapy; Individual	Contents for 8 weeks included: (examples only provided) (1) Assessing the situation of empty nest elderly; (2) Introducing reminiscence therapy; (3)-(7) Reminiscence activities; (8) Looking to the future.	RCT 8 sessions, 45-60 min; weekly; Not mentioned	Usual care; Senior nurse	1. Depression(GDS) 2. Happiness(MUNSH)
Wu (2010)	Taiwan, China; Veterans' homes	81.34 (SD=3.34) (0%)	74 35/39	Integrative reminiscence therapy; Group	Topics for 12 weeks included: (examples only provided) Introduce myself; Past photos I missed; A memorable trip; A most memorable person; Proud things in life; Present life.	Quasi-experimental Study,12 sessions,1 hr each,weekly; Not mentioned	Daily home activities; Director	1. Life satisfaction(LSI-A) 2. Self-esteem (SES) 3. Depressive symptoms (GDS-SF)
Yousefi (2015)	Iran; Day care center	⩾ 60 (Mean age not reported) (100%)	29 14/15	Narrative reminiscence therapy; Group	Topics for 3 weeks included: (1) Major decisive events of life; Family life; (2) Career or major life work and personal interests; Stress experiences; (3) Loves and hates; Beliefs on the meaning and goals of life.	RCT 6 sessions, 1.5-2 hr each; twice a week; 1-month follow-up	Participated group discussions; Group facilitator	1. Happiness (OHQ)

Abbreviations:ASADE, Assessment Scale of Adaptation Difficulty for the Elderly; AES, Apathy Evaluation Scale; ACE-III, Addenbrooke Cognitive Examination III; BRCS, Brief Resilient Coping Scale; CAE, Coping Strategies Questionnaire; GSE, General Self-Efficacy Scale; GDS, Geriatric Depressive Scale; LIS, Life Integration Scale; LGS, Loyola Generativity Scale; LSI-A, Life Satisfaction Index A; MSEQ, Memory Self-Efficacy Scale; MMSE, Mini-Mental State Examination; MUNSH, Scale of Happiness of the Memorial University of Newfoundland; NPS, Neuropsychiatric symptoms; OHQ, Oxford happiness questionnaire; PGCMS, Philadelphia Geriatric Center Morale Scale; PSS, Perceived Stress Scale; QLI, Quality of Life Index; RSES, Rosenberg Self-Esteem Survey; RULS-V3, Revised University of California Los Angeles loneliness scale; RFS, Reminiscence Functions Scale; SPWB, Psychological Well-Being Scales; STAI, Spielberger Anxiety Inventory; SCL-90-R, Symptoms checklist-90-R; SALSS, Self-Anchoring Life Satisfaction Scale; UCLA, UCLA loneliness scale.
